# Contemporary Use of Polymers in Dentistry: A Narrative Review

**DOI:** 10.3390/polym18010138

**Published:** 2026-01-02

**Authors:** Svetla Ivanova, Zlatina Tomova, Angelina Vlahova, Iliyana L. Stoeva, Elena Vasileva, Yordanka Uzunova, Magdalina Urumova, Desislav Tomov, Atanas Chonin

**Affiliations:** 1 Department of Prosthetic Dentistry, Faculty of Dental Medicine, Medical University of Plovdiv, 15-A “Vasil Aprilov” Blvd, 4002 Plovdiv, Bulgaria; zlatina.tomova@mu-plovdiv.bg (Z.T.); angelina.vlahova@mu-plovdiv.bg (A.V.); elena.vasileva@mu-plovdiv.bg (E.V.); magdalina.urumova@mu-plovdiv.bg (M.U.); 2 Department of Imaging Diagnostics, Dental Allergology and Physiotherapy, Faculty of Dental Medicine, Medical University of Plovdiv, 15-A “Vasil Aprilov” Blvd, 4002 Plovdiv, Bulgaria; iliyana.stoeva@mu-plovdiv.bg (I.L.S.); atanas.chonin@mu-plovdiv.bg (A.C.); 3 Department of Bioorganic Chemistry, Faculty of Pharmacy, Medical University of Plovdiv, 15-A “Vasil Aprilov” Blvd, 4002 Plovdiv, Bulgaria; yordanka.uzunova@mu-plovdiv.bg; 4 Research Institute at Medical University of Plovdiv, Department of Bioorganic Chemistry, Faculty of Pharmacy, Medical University of Plovdiv, 15-A “Vasil Aprilov” Blvd, 4002 Plovdiv, Bulgaria; desislav.tomov@mu-plovdiv.bg

**Keywords:** dental polymers, denture base materials, restorative materials, impression materials, 3D printed resins

## Abstract

This narrative review examines contemporary applications of polymeric materials in dentistry from 2020 to 2025, spanning prosthodontics, restorative dentistry, orthodontics, endodontics, implantology, diagnostics, and emerging technologies. We searched PubMed, Scopus, Web of Science, and Embase for peer reviewed English language articles and synthesized evidence on polymer classes, processing routes, mechanical and chemical behavior, and clinical performance. Approximately 116 articles were included. Polymers remain central to clinical practice: poly methyl methacrylate (PMMA) is still widely used for dentures, high performance systems such as polyether ether ketone (PEEK) are expanding framework and implant-related indications, and resin composites and adhesives continue to evolve through nanofillers and bioactive formulations aimed at improved durability and reduced secondary caries. Thermoplastic polyurethane and copolyester systems drive clear aligner therapy, while polymer-based obturation materials and fiber-reinforced posts support endodontic rehabilitation. Additive manufacturing and computer aided design computer aided manufacturing (CAD CAM) enable customized prostheses and surgical guides, and sustainability trends are accelerating interest in biodegradable or recyclable dental polymers. Across domains, evidence remains heterogeneous and clinical translation depends on balancing strength, esthetics, biocompatibility, aging behavior, and workflow constraints.

## 1. Introduction

Polymers rank among the most extensively used materials in dentistry, offering essential applications across nearly every dental specialty. The versatility of polymeric materials—ranging from hard, rigid structures to soft, elastomeric forms—allows them to serve diverse functions such as replacing missing tissues, bonding to tooth structures, or delivering therapeutic agents [1].

A polymer is a macromolecular substance formed by the repetition of structural units (monomers) linked by covalent bonds; in dentistry the term ‘polymer’ typically refers to synthetic or natural materials composed of long molecular chains [2].

Polymers are typically classified into two groups: synthetic polymers, such as polyolefins, fluorinated polymers, polyesters, and silicones, and natural polymers, including polysaccharides and proteins [3].

These materials appear in items of everyday dentistry: denture bases, composite fillings, adhesives, sealants, impression materials, clear aligners, posts, and more. Key advantages of polymers in oral applications are their biocompatibility and tunable properties. Polymers can often be formulated or modified to achieve desirable characteristics such as flexibility or rigidity, transparency, pigmentation to match tooth or gum color, and controlled setting times. Unlike metals or ceramics, many polymers are lightweight and can be processed at lower temperatures or via additive manufacturing, enabling chairside or laboratory fabrication of customized appliances. Furthermore, polymers generally pose low risk of systemic toxicity or allergic reaction when properly cured, as they can be made with minimal leachable components. This makes them well-suited for intraoral use, provided that any residual monomers or additives are minimized to prevent irritation [1].

However, limitations persist with traditional dental polymers, necessitating continuous innovation. For example, the methacrylate-based resins widely used in dentures and obturations tend to absorb water and undergo polymerization shrinkage or hydrolytic degradation over time, potentially compromising their fit or strength. Many polymers are also softer than enamel or ceramics, which can increase susceptibility to wear in the harsh oral environment over years. Discoloration, however, is primarily linked to water sorption and surface adsorption of pigments, as well as surface roughness, and these factors are strongly influenced by resin matrix composition and finishing and polishing protocols [4,5,6]. Consequently, recent research (2020 onward) has been devoted to enhancing polymer performance, e.g., through nanoparticle reinforcement, novel monomer chemistries to reduce shrinkage, and polymer blends or fibers that improve mechanical properties [7].

To reduce bacterial and fungal colonization on PMMA, various active nanoparticles can be integrated into its structure before the final polymerization. Common examples include zirconium dioxide (ZrO_2_-NPs), silver (Ag-NPs), and platinum (Pt-NPs) [8].

One of the fastest-growing innovations in dentistry is additive manufacturing, a technique that allows clinicians to create highly accurate, complex restorations and structures from virtual designs. Depending on the intended use, various 3D printers and polymers are utilized, such as polycarbonates, polylactic acid, polyurethanes, polyether ketone ketone, polylactic-co-glycolic acid, polyether ether ketone, polyvinyl alcohol, polycaprolactone, poly-glycolic acid, polybutylene terephthalate, acrylonitrile butadiene styrene, and polymethyl methacrylate [8,9].

The present narrative review provides an up-to-date overview of polymer use in dentistry, structured by major application areas. For each domain—prosthodontics, restorative dentistry, orthodontics, endodontics, implantology, diagnostics, and emerging technologies—we summarize the dominant polymer types, their properties, advantages and drawbacks, and recent innovations/improvements. Comparative tables are included to highlight key polymers, their applications and relevant properties (mechanical, chemical, biological), as reported in current literature. By synthesizing findings from 2020 onwards, the review aims to inform clinicians and researchers of the current state-of-the-art in dental polymers and anticipated future directions, including the push towards bioactive and sustainable materials.

## 2. Materials and Methods

This narrative review used a structured literature search and selection process to identify representative and clinically relevant studies; it was not conducted as a formal systematic review.

Search Strategy: Electronic searches were performed in four databases (PubMed/Medical Literature Analysis and Retrieval System Online (MEDLINE), Scopus, Web of Science, and Embase) using combinations of keywords such as “dental polymer”, “dentistry materials polymer”, “resin composite”, “denture base polymer”, “orthodontic polymer”, “PEEK dentistry”, “3D printing dental resin”, and specific terms for each specialty (e.g., “orthodontic aligner material”, “endodontic post fiber polymer”). The search was restricted to peer-reviewed articles in English published from 2020 onwards. Additional manual search was performed in reference lists of pertinent articles and within specialty journals (e.g., Dental Materials, Journal of Prosthodontics, Clinical Oral Investigations) to ensure comprehensive coverage.

Inclusion and Exclusion Criteria: Original articles were included, systematic or narrative reviews, and relevant case series that focused on the use or evaluation of polymeric materials in any dental field. Articles had to present data on material properties, clinical performance, or technological developments of polymers. Studies purely about historical materials (pre-2020) or not offering insight into current materials were excluded, and so were those not specific to dental applications. Where multiple studies covered similar content, preference was given to the most recent or comprehensive source.

## 3. Results

### 3.1. Prosthodontics

Prosthodontics encompasses the fabrication of dental prostheses such as complete and partial dentures, crowns, bridges, and maxillofacial prosthetics. Polymeric materials have long been central in this field and continue to evolve to meet functional and esthetic demands. Table 1 summarizes key polymers used in prosthodontics, their applications, and pertinent properties.

Polymethyl methacrylate (PMMA) remains the standard material for denture bases due to its favorable processing characteristics, aesthetics (gum-colored translucency), adequate strength, and low cost. Conventional heat-cured PMMA offers good initial mechanical properties but can be prone to fracture after years of service, in part due to its rigidity and internal stresses [1].

Numerous strategies are reported to improve PMMA performance, such as chemical modifications and reinforcement with fibers or nanoparticles. For instance, adding glass or polyaramid fibers, or nano-fillers like zirconia or silica, can increase the flexural strength and impact resistance of PMMA denture resin. PMMA can also be formulated as high-impact resin with rubbery co-polymers to enhance toughness. Despite these enhancements, PMMA’s inherent limitations (polymerization shrinkage, residual monomer causing tissue irritation, and candida biofilm accumulation) drive ongoing research. Antimicrobial modifications, such as incorporating silver or zinc-oxide nanoparticles into denture resin, have demonstrated reduced biofilm formation on denture surfaces. Similarly, surface coatings with biocompatible polymer films (e.g., phospholipid polymers) can make the denture base more resistant to bacterial adhesion [9,10].

An important recent trend is the use of digital fabrication for dentures. CAD/CAM milled PMMA denture bases (from pre-polymerized PMMA blocks) show higher density, improved fit, and often greater strength than traditional flask-processed ones [11].

Milled PMMA bases eliminate the polymerization shrinkage issue (since the material is already cured), yielding better adaptation. Likewise, rapid prototyping methods (3D printing) now allow direct fabrication of denture bases from light-cured resin formulations. These printable denture base resins are typically methacrylate-based but specially tuned for photopolymerization. Early studies indicate printed dentures have acceptable accuracy and properties, though slightly lower strength than milled bases, and research is ongoing to improve printed resin chemistries [12].

Overall, digital techniques expand the polymer options (e.g., use of crosslinked acrylic resins or composite resins for printing) and enable quicker production of prostheses.

Polyetheretherketone (PEEK)-based dental prostheses represent a promising alternative to conventional materials due to their excellent biocompatibility, favorable patient acceptance, and strong mechanical properties. Nonetheless, additional research is necessary to evaluate their long-term outcomes and success rates. From a biocompatibility standpoint, PEEK demonstrates outstanding compatibility with biological tissues, making it well-suited for dental applications. Moreover, studies have confirmed that PEEK is non-mutagenic and non-cytotoxic, supporting its safe use in the oral environment [13].

Patients generally respond well to PEEK-based dental prostheses, appreciating their natural esthetics and metal-free composition. As implant-supported restorations become a popular solution for Treatment of partial edentulism PEEK is gaining attention as a material of choice for a range of implant prostheses [14].

The surface properties of PEEK are crucial for its polishability and influence bacterial adhesion, an important factor in prosthetic applications. PEEK exhibits excellent polishing potential, which helps minimize bacterial colonization and plaque formation. Surfaces polished to a roughness (Ra) below 0.13 µm are considered optimal for reducing bacterial adhesion [15]. Glazing PEEK surfaces effectively improves surface smoothness, independent of artificial aging or staining procedures [16].

Compared to milled, injection-molded, or 3D-printed PEEK, pressed PEEK exhibited higher levels of bacterial adhesion. Horizontally printed PEEK showed better resistance to bacterial buildup than vertically printed samples. Maintaining surface roughness under 10 µm or performing additional polishing seems vital to further reduce bacterial adhesion on PEEK [17].

Silicone elastomers (especially medical-grade polydimethylsiloxane) are the dominant polymers for facial prostheses (nose, ear, etc.) because of their flexibility and skin-like feel. They can be colored intrinsically or extrinsically to match skin tones. Recent advances include nanosilica filler reinforcement to improve tear strength of facial silicones and pigments with better ultraviolet (UV) stability to reduce color degradation over time (a common issue for facial prostheses exposed to sunlight). Intraorally, soft denture liners utilize silicone or plasticized acrylic polymers. These liners cushion the interface between a hard denture base and the mucosa, beneficial for patients with sharp bony ridges or sore spots. Silicones offer long-term softness and bioinertness, whereas acrylic (polyethyl methacrylate with phthalate plasticizers) liners provide softness initially but may harden with time as plasticizer leaches. Research in the past few years has introduced silicone liners containing antimicrobial agents (e.g., silver nanoparticles or ammonium compounds) to resist fungal colonization (denture stomatitis). Another niche prosthodontic polymer is polyamide (nylon) used in “flexible” removable partial dentures. These thermoplastic nylon baseplates are unbreakable and metal-free, with flexible clasps that adapt to tooth undercuts. They have no residual monomer and are indicated in cases of allergy to PMMA [18].

However, polyamides can absorb water and stain, and their difficulty in adjustment and relining has limited widespread use.

Provisional Crowns and Bridges: For interim prostheses, polymer-based materials are standard. Bis-acrylic composite resins (two-component materials that self-cure into a resin with fillers) are popular for chairside fabrication of temporary crowns/bridges due to their ease of use and better wear resistance than older polymethyl methacrylate temps. CAD/CAM PMMA blocks are also used to mill long-term temporaries; they possess high strength and can serve for extended time (months) during implant osseointegration in complex rehabilitation. Some high-impact polymers like PEKKTON (polyetherketoneketone-based high performance polymer from Cendres Metaux) have been explored for long-term provisional implant-supported crowns because of their toughness and retrievability [12,19].

A study comparing the wear resistance, abrasiveness, color stability, and displacement resistance of zirconia and PEEK milled crowns found that PEEK crowns exhibited minimal abrasion, superior stress distribution through plastic deformation, and stable color properties, positioning PEEK as a promising alternative to zirconia for crown fabrication [20].

Polymers remain fundamental to prosthodontic applications, offering versatile solutions for denture bases, frameworks, provisional restorations, and maxillofacial prostheses. PMMA continues to dominate denture fabrication, although its inherent limitations have driven innovations such as fiber reinforcement, nanoparticle incorporation, and digital fabrication through CAD/CAM milling and 3D printing. High-performance polymers like PEEK are gaining interest due to their superior mechanical properties, biocompatibility, and patient acceptance, particularly in implant-supported prostheses. Silicone elastomers and polyamides also play key roles in facial prosthetics and flexible partial dentures, although challenges like material aging and water absorption persist. Overall, advances in polymer chemistry, manufacturing techniques, and antimicrobial modifications are expanding the capabilities of prosthodontic polymers, improving clinical outcomes and patient satisfaction.

The main polymeric materials used in prosthodontics, together with their key properties and clinical applications, are summarized in Table 1.

**Table 1:** Polymers in Prosthodontics and Their Properties.

Polymer	Prosthodontic Applications	Key Properties	Advantages	Limitations
Poly(methyl methacrylate) (PMMA)	Denture bases (complete/partial dentures); denture teeth; provisional crowns	Rigid thermoplastic, Tg ~105 °C. Flexural strength ~80 MPa; Modulus ~2–3 GPa. Some water sorption and moderate polymerization shrinkage [9,18].	Easy to process (mold or CAD/CAM); inexpensive; color match to gums/teeth; fair strength and repairable	Brittle (fracture risk); residual monomer can irritate tissues; prone to bacterial/fungal colonization without additives
Polyetheretherketone (PEEK)	Frameworks for removable partial dentures; implant-supported bridge frameworks; implant abutments; provisional implant crowns	High-performance semi-crystalline polymer, Tg ~143 °C Tensile strength ~90–100 MPa; Young’s modulus ~3.5–4 GPa (closer to bone). Highly chemical- and wear-resistant [13,14,15,16,17,19].	Very strong and fatigue-resistant; lightweight; biocompatible (no monomer, low allergy); radiolucent; shock-absorbing elasticity; can be milled with CAD/CAM	Opaque/esthetic limitations (requires veneering for visible areas); inherently hydrophobic (difficult to bond, needs surface treatment); material cost is high; equipment needed for milling.
Polyamide (Nylon)	Flexible partial denture bases and clasps (“nylon dentures”)	Semi-crystalline thermoplastic. Lower flexural modulus (~1–2 GPa). High elongation at break (flexible) [12,18].	Unbreakable (very high impact strength); flexible clasps for undercuts; monomer-free (good for patients with allergies)	Difficult to adjust or reline; can absorb water and stain; less dimensional stability.
Silicones (PDMS-based)	Maxillofacial prostheses (ear, nose, etc.); soft liners for dentures; occlusal guards/nightguards	Elastomeric polymer with low modulus. Tear strength varies (~5–10 kN/m for reinforced maxillofacial silicones). Durable up to ~200 °C [18].	Excellent biocompatibility; flexible and soft (patient comfort); colorable for lifelike appearance; chemically inert in mouth.	Susceptible to fungal growth in liners (unless treated); maxillofacial silicone colors can fade under UV; lower tear resistance than ideal (prone to edge tears).
Bis-acrylic composite resin	Provisional crowns and bridges (direct chairside temporaries)	Cross-linked polymer matrix with filler (similar to composite). Compressive strength ~200–300 MPa. Moderately hard, some wear over weeks-months [12,20].	Fast and easy auto-curing; improved wear and aesthetics vs. older acrylic temps; low heat release during set.	Brittleness over long term; not for permanent use (can fracture under heavy load); limited shade range and polish compared to ceramics.

Sources for numerical values in table: [9,12,13,14,15,16,17,18,19,20].

Clinical relevance: PMMA remains the default denture base and provisional material, while CAD CAM workflows and high-performance polymers such as PEEK expand options for long term provisionals, implant provisionalization, and metal-free frameworks when bonding and surface finishing protocols are followed [9,12,15,18].

Limitations and evidence gaps: Evidence is still dominated by in vitro studies and short follow up cohorts, and outcomes are sensitive to processing route, surface treatment, and antagonist material; long term comparative clinical trials for PEEK and printed resins remain limited [15,18].

### 3.2. Restorative Dentistry

Restorative dentistry heavily relies on polymer-based materials for direct restorations, indirect restorations, and adhesive bonding systems. The advent of polymeric composite resins revolutionized tooth-colored restorations, and continuous improvements are being made to address their shortcomings (like shrinkage and wear). Dental composites, adhesives, sealants, and cements are the main polymer-containing restoratives, often based on methacrylate monomers. Key developments since 2020 include enhancing the bioactivity of restoratives to combat recurrent caries and refining resin formulations for bulk placement and durability.

The modern era of restorative polymers began with the introduction of Bis-GMA (bisphenol A glycidyl methacrylate) by Dr. Rafael Bowen in the 1960s, establishing the foundation for today’s resin matrices. Early composites significantly excels PMMA by offering lower polymerization shrinkage, greater mechanical strength, better thermal expansion matching with tooth structures, and the possibility of reliable adhesion via the acid etching technique pioneered by Dr. Michael Buonocore [21,22].

Today’s universal direct filling materials are typically bis-GMA (bisphenol A-glycidyl methacrylate) or similar dimethacrylate matrix composites loaded with silica or ceramic fillers (50–80% by volume). These composites offer excellent initial esthetics and adequate strength for anterior and posterior use, but they are not without issues. Polymerization shrinkage (typically 2–3% volumetric) can induce stress at the tooth-restoration interface, potentially leading to debonding or microleakage. Additionally, the resin matrix can absorb water and undergo hydrolysis over time, softening the material [23].

To tackle these issues, recent composites have focused on shrinkage reduction and enhanced mechanical performance. Novel low-shrink monomers (such as siloranes or dimer-acid-based methacrylates) were introduced to reduce stress, though adoption is limited and most current products still use methacrylate chemistry. The development of bulk-fill composites, which allow curing in 4–5 mm increments thanks to advanced photoinitiators and higher translucency, has significant impact on dental treatment. These materials often include urethane dimethacrylate derivatives (UDMA) that exhibit lower polymerization stress. Nano-hybrid and nano-filled composites (with filler particles < 100 nm) have become prevalent, offering a good balance of strength and polishability. A 2021 study of modern nanocomposites showed improved wear resistance and reduced water sorption compared to earlier hybrid composites, attributing this to better coupling and higher filler content [24].

The development of bioactive restorative materials is another significant trend. These are polymeric composites or glass-ionomer hybrids designed to release therapeutic ions (fluoride, calcium/phosphate) or have antibacterial effects. For example, giomer composites incorporate pre-reacted glass filler that can release fluoride, while some composites include calcium silicate or phosphate fillers to promote remineralization of adjacent tooth structure. There has also been intense research on antimicrobial monomers and additives. Quaternary ammonium methacrylates (QAMs) can be copolymerized into adhesives or composites to endow them with contact-killing abilities against bacteria [25,26,27].

Many additives can reduce composite strength or adversely affect color/transparency if not optimized. Nonetheless, some commercial “bioactive composites” and sealants have appeared, though systematic reviews thus far indicate they have no significant clinical advantage yet in preventing secondary caries compared to traditional composites [28].

Although conventional composites remain the most widely used resin-based materials in clinical dentistry, CAD/CAM resin-based composite blocks are gaining popularity as a reliable restorative alternative. These CAD/CAM composite blocks are generally divided into two groups. The first group is resin composite blocks RCBs. The second group is the polymer infiltrated ceramic network (PICN) [29].

CAD CAM resin composite blocks exhibit a high degree of conversion because their polymerization is carried out under high pressure and high temperature. This manufacturing route produces a more homogeneous composite with fewer defects and pores than conventional indirect composites and it also enables the use of a higher filler load. These materials therefore demonstrate better mechanical behavior including increased wear resistance, greater flexural strength, and higher fracture toughness and fracture strength compared with traditional indirect resin composites.

Polymer infiltrated ceramic network (PICN) materials are fabricated by first creating a porous pre-sintered ceramic network that is treated with a coupling agent and then infiltrated with a polymer. As a result, PICN possesses a three-dimensional ceramic scaffold that improves resistance to structural breakdown [30,31]. The presence of ceramic matrix provides the good resistance to wear. The combined structure of ceramics and polymer has a preventive effect on the spread of cracks and fractures within the restorations. Since this is a recently introduced material, there are still no clinical studies that follow PICN restorations over a long time. Its shade selection is limited, and there is still no reliable evidence for its performance in cervical regions or for resistance to discoloration. Current indications include veneers, inlays and onlays, anterior and posterior single crowns, and implant supported prostheses. However, it is better suited for restorations in the posterior area because its esthetic potential is lower than that of other options [32].

According to Paolone et al. CAD CAM blocks demonstrate better color stability than direct or indirect laboratory resin-based composites although they still do not reach the color stability of ceramic materials. Their color stability is determined mainly by the material composition and by the staining medium and it is further influenced by the finishing and polishing procedure [33].

Dental sealants placed on primary and permanent teeth serve as a protective layer that isolates the pits and fissures from the oral environment. Methyl cyanoacrylate was the first material used for this purpose in the 1960s by Cueto, but it was gradually broken down by bacteria over time. Later, Bowen introduced a thicker resin called BIS GMA that could bond to etched enamel and that did not show the same bacterial degradation as Cueto’s material [34].

One such approach is the use of dental sealants applied to the chewing surfaces of teeth to help prevent caries formation. The proven effectiveness of sealants has significantly contributed to lowering caries incidence rates. Enhancing sealant materials to become biointeractive presents a valuable opportunity for caries prevention. This way they not only may act as a physical barrier against food accumulation but would actively contribute to the enamel integrity and health. Since sealants remain in close contact with enamel areas prone to decay, they can serve as ideal carriers for bioactive components such as nanoparticles of amorphous calcium phosphate (NACP). This concept has led to the development of rechargeable antibacterial sealants containing NACP, which hold promise for preventing enamel demineralization and promoting remineralization [35].

Several materials are now used as pit and fissure sealants, mainly resin-based sealants (RBS) and glass ionomer (GI) sealants. Resin-based sealants are grouped into four generations according to how they polymerize. Nuva Seal represents the first generation and was cured with ultraviolet light, but it is no longer in use. The second generation is set chemically through the addition of a tertiary amine. The third generation is cured by light and therefore has a short setting time. The fourth generation consists of fluoride releasing RBS. Depending on how viscous they are, resin-based sealants can be either filled or unfilled, and they can also be produced as opaque or as transparent materials [36].

An overview of the polymeric materials currently used in restorative dentistry and their primary characteristics is presented in Table 2.

Clinical relevance: Resin-based composites remain the primary materials for direct restorations. Newer formulations and CAD CAM composite blocks can reduce chair time and improve handling, but clinical success still depends on isolation, incremental strategy when indicated, and adequate curing [21,22,29].

Limitations and evidence gaps: Polymerization shrinkage stress, hydrolytic degradation, and surface staining continue to drive marginal breakdown and replacement. Many innovations are supported mainly by laboratory outcomes, and well powered head-to-head clinical trials with standardized endpoints remain scarce [22,33].

### 3.3. Orthodontics

Polymeric materials have become increasingly important in orthodontics, particularly with the rise in clear aligner therapy and other esthetic appliance systems. Traditional fixed braces also utilize polymers in various forms (elastic ligatures, bonding adhesives), but the past decade’s surge in demand for nearly invisible orthodontic treatments has driven rapid development of specialized polymers. Table 3 summarizes key polymer applications in orthodontics.

Clear Aligners: Introduced in the late 1990s, clear aligners (Invisalign and others) are now a mainstream orthodontic option. These removable appliances are essentially thin polymer trays that incrementally move teeth. The polymers used must be transparent, biocompatible thermoplastics that can sustain continuous mechanical forces. Early aligners were often single-layer materials like polyurethane. Modern aligner systems use various thermoplastic polymers, commonly thermoplastic polyurethanes (TPU), polyethylene terephthalate glycol (PETG), and other copolyesters [37].

These materials are usually supplied as flat sheets which are thermoformed over a dental model to create the aligner. Key properties include moderate stiffness to apply force, high flexibility and resilience (to snap over teeth without cracking), and optical clarity [38,39].

One challenge is that aligners undergo mechanical and chemical aging in the mouth—exposure to saliva, temperature changes, and stresses may cause degradation of mechanical properties over the 1–2 weeks an aligner is worn. For instance, some co-polyesters can undergo hydrolysis (especially in a warm, wet environment), becoming slightly more brittle or deforming. TPU materials, which contain hard and soft segments, may absorb some water and creep under constant stress, potentially reducing the effective force over time. To counteract these issues, companies have developed multi-layer aligner materials combining different polymers (for example, a stiffer inner layer providing force, and a softer outer layer providing comfort and stain resistance). Research indicates that such multi-layer designs can better maintain forces over the wear period and improve patient comfort by reducing initial force peaks. Additionally, to improve optical clarity and stain resistance, new thermoplastics like aromatic copolyesters and medical-grade polycarbonates have been tested. Aligners must also be safe—studies confirm that quality aligner polymers do not leach harmful substances at significant levels and are generally non-toxic [40].

A very recent innovation is the use of shape-memory polymers (SMPs) for aligners. SMPs can be programmed to change shape in a controlled manner upon a stimulus (like heat). In orthodontics, an SMP aligner could theoretically apply a more constant force or even adjust itself gradually, reducing the number of different aligners needed [41].

Advances and innovations in the field of orthodontic aligners have been primarily directed toward enhancing the efficiency of fabrication and clinical workflows, while simultaneously reducing the overall treatment time and associated costs. Notably, the incorporation of digital technologies into the production of orthodontic aligners represents one of the most transformative developments in orthodontics in the 21st century [42].

A wide range of 3D printers, thermoforming machines, and plastic foils with diverse specifications and functionalities are currently available on the market. Concurrently with the rapid progress of digital technologies, 3D printing systems have also advanced, enabling the fabrication of a variety of resin-based materials [41].

Printed aligners could reduce material waste (no discarded plastic sheet margins or plaster models) and simplify the workflow. However, to date, only a few materials meet the necessary transparency, strength, and biocompatibility standards for direct-printed aligners. The field anticipates more such materials emerging, with careful evaluation needed for their mechanical consistency and safety (uncured resin residues must be minimized) [38,41,43].

In fixed braces, elastic ligatures (the small colored rubber bands that hold arch wires to brackets) are typically made of polyurethane elastomer. Latex rubber bands are another polymeric item (natural polyisoprene) still widely used for inter-arch traction.

Bonding brackets to teeth relies on composite resin adhesives, akin to those in restorative dentistry. Typically, a lightly filled Bis-GMA resin is used with a quick cure. These have been refined to provide adequate working time and a strong bond to often non-ideal surfaces (sometimes bonding to porcelain crowns or composite fillings in adult patients).

While most orthodontic archwires are metal (NiTi, stainless steel), there are polymer-related innovations. Coated archwires with aesthetic tooth-colored polymer coatings (e.g., Teflon or epoxy resin) are used for patients desiring less visible appliances. These coatings can wear off, but improvements aim to make them more durable and stain-resistant. Fully polymeric archwires are not common due to insufficient stiffness, but fiber-reinforced composite archwires have been explored. These involve embedding glass or polyaramid fibers in a resin matrix to create a wire-like rod. They can be made to roughly match the force of a light NiTi wire and are nearly invisible. However, their use has been limited to date, partly because tying them in and achieving consistent forces is challenging [44,45].

After orthodontic treatment, removable retainers (like Essix retainers) are also made of thermoplastic polymers, often similar to aligner materials (PETG or polypropylene sheets). These are usually tougher/thicker than aligners since they must hold teeth in place long-term. Thermoplastic mouthguards for sports or for habit intervention (tongue thrust appliances, etc.) are yet another application, typically produced from ethylene vinyl acetate or polyurethane sheets [46,47,48].

The main polymer based materials used in orthodontics, including aligner materials and their mechanical characteristics, are summarized in Table 3.

**Table 3:** Polymers in Orthodontics.

Polymer/Material	Orthodontic Use	Properties	Notes on Performance
Thermoplastic Polyurethane (TPU)	Clear aligners (e.g., Invisalign); power chain elastics (polyurethane elastomer)	TPU aligner: transparent, Shore hardness ~80–90 A. Exhibits elastic recovery but some force decay over 1–2 weeks Power chain: high elasticity, gradual force loss as polymer relaxes [38,39].	TPU aligners provide flexibility and toughness; newer formulations maintain strength longer under oral conditions. Often used in multi-layer aligner sheets for balanced force and comfort.
PETG (Polyethylene terephthalate glycol-modified)	Clear aligners and retainers (thermoformed sheets)	Amorphous copolyester, clear. Higher initial stiffness than TPU, with elastic modulus ~1.5–2 GPa. Can undergo hydrolytic degradation if exposed to heat/moisture over time [38,39].	Provides strong initial force in aligners; may be more prone to stress cracking if overstressed. Common in many aligner systems (often marketed under trade names).
Polycarbonate or Copolyester blends	Aesthetic brackets; some aligner materials	Rigid, transparent polymers. Used in “clear” brackets (polycarbonate brackets)—modulus ~2–3 GPa. In aligner context, added for rigidity in layered materials [37,39].	Polycarbonate brackets are less brittle than ceramic but can stain and deform; primarily used in low-load situations (they can have higher friction in sliding mechanics). In aligners, these polymers add stiffness and are usually sandwiched with TPU layers.
Silicone elastomer	Orthodontic elastics (some intra-oral elastics for latex-free option)	Soft, rubber-like; high elasticity. Not as common as latex for interarch elastics (lower modulus, larger extension needed).	Typically used when latex allergy is a concern. Force decay tends to be higher than latex over time.
Resin adhesive (light-cure orthodontic composite)	Bonding brackets and appliances to teeth	Bis-GMA/Bis-EMA resin with filler (~40–60% wt). High bond strength to enamel (often 15–25 MPa). Fluoride-releasing versions exist [40,44].	Must be durable in presence of orthodontic forces. Newer adhesives minimize bracket failure and help prevent white spots via fluoride release or antimicrobial agents. Debonding at case end can be done by resin fracture or softening (thermally or with laser) of this polymer.
Shape-memory polymer (experimental)	Next-generation aligners (programmed force delivery)	Polymer with dual-segment molecular architecture enabling shape change when triggered (e.g., warming to mouth temperature).	In development: aim to reduce number of aligners needed by having one appliance that gradually adjusts shape. Need to ensure predictable tooth movement and biocompatibility.

Sources for numerical values in table: [37,38,39,40,41,44].

Clinical relevance: Thermoplastic polymers enable clear aligners and a range of removable appliances. Force delivery and fit retention are governed by viscoelastic relaxation, thermoforming parameters, and oral aging; material selection and thickness should align with the intended force system [38,40,47].

Limitations and evidence gaps: Much of the evidence comes from short term mechanical testing, while real world performance is affected by temperature cycling, saliva exposure, and patient wear behavior. More longitudinal clinical studies linking polymer aging to tooth movement predictability are needed [38,40].

### 3.4. Endodontics

The filling of root canals, known as obturation, is a vital component of endodontic therapy. The procedure aims to achieve a tight apical seal and to densely fill the root canal system without voids. Proper obturation is essential for preventing the ingress of bacteria, saliva, and periapical fluids, as well as for entombing any residual microorganisms within the canal, thereby minimizing the risk of reinfection [49].

Currently, a wide range of root canal sealers is available, encompassing zinc oxide eugenol, glass ionomer, calcium hydroxide, silicone-based, epoxy resin, tricalcium silicate (MTA/bioceramic), methacrylate resin, and bioceramic-based formulations [50].

Among the various obturation techniques, cold lateral condensation and vertical condensation of thermoplasticized gutta-percha remain the most widely used. Lateral condensation is often regarded as the benchmark for assessing newer methods and is preferred for obturating teeth with open apices. The heated gutta-percha technique was introduced to improve material adaptation to canal irregularities and minimize voids; however, in vitro studies have not consistently validated these advantages. While thermoplasticized techniques may produce fewer voids, the sealing of isthmuses and lateral canals still largely depends on the sealer [51].

Resilon is a thermoplastic synthetic material intended for root canal obturation, composed primarily of polycaprolactone (PCL), a biodegradable aliphatic polyester. Its filler system includes bioactive glass, bismuth oxychloride, and barium sulfate, contributing to its enhanced radiopacity relative to dentin. The polymeric matrix contains approximately 25–40% PCL and 3–10% dimethacrylate components. The accompanying sealer is a semi-crystalline polymer with a melting point near 60 °C, capable of releasing Na^+^ and Ca^2+^ ions. Resilon’s tensile strength, elastic modulus, and thermal properties closely resemble those of gutta-percha, promoting dimensional stability post-setting. Notably, Resilon exhibits greater thermoplastic behavior than other thermoplastic gutta-percha-based systems such as Endoflow and Obturaflow [52].

Sealers fill the space between the gutta-percha (or other cores) and canal wall. There are several classes, many of which are polymeric: epoxy resin-based sealers (e.g., AH Plus), methacrylate resin-based sealers (like EndoREZ), silicone-based sealers, etc. [49].

Epoxy resin-based sealers were first introduced into endodontic practice by Schroeder, and modern adaptations of the original formulation remain widely utilized in root canal obturation today.

AH Plus is a widely used epoxy resin-based root canal sealer known for its excellent sealing ability, low solubility, and dimensional stability. It polymerizes to form a crosslinked network, providing long-term sealability. However, it relies on mechanical adaptation rather than chemical bonding to dentin [53].

A methacrylate-based, dual-cure resin sealer has been introduced for root canal obturation to improve adaptation between the filling material and dentinal walls. This hydrophilic system is capable of penetrating dentinal tubules and forming resin tags, which contribute to micromechanical retention and enhanced sealing performance. Recent studies further confirm its ability to infiltrate dentin and create a tight interface while maintaining adequate film thickness and acceptable physicochemical properties [54,55].

After root canal treatment, if the tooth is structurally compromised, a post is often placed to retain a core buildup.

Although the restoration of endodontically treated teeth is a routine aspect of restorative dentistry, there remains a considerable debate regarding the optimal choice of materials and techniques. The drawbacks associated with metallic posts-including risks of corrosion, root fractures, and retention failure combined with the growing demand for highly aesthetic restorations, have driven the development of posts made of aesthetic materials such as zirconia ceramics, fiber-reinforced composites, and polyetherketoneketone (PEKK) [56].

The rising emphasis on aesthetics in dentistry has led to a growing preference for non-metallic posts over traditional metallic ones. Their elastic modulus, closely matching that of natural dentin, contributes to a reduced risk of vertical root fractures [57,58].

Fiber posts are typically classified as composite materials comprising three distinct phases: the continuous resin matrix, the dispersed reinforcing fibers, and the interphase region that mediates the interaction between the two [59].

Fiber-reinforced composite posts (FRCPs) are fabricated using different types of fibers—such as carbon, polyaramid, polyethylene, and glass—embedded within a variety of resin matrices. These matrices include epoxy-based systems, cross-linked dimethacrylate resins, Bis-GMA formulations, and, less commonly, aromatic polyimides [56].

Although continuous fiber composites utilizing thermoplastic polymer matrices have not been extensively applied in dental practice, they are regarded as advanced structural materials. They are characterized by favorable mechanical performance, ease of fabrication, low density, recyclability, cost-efficiency, and exceptional resistance to corrosion [60].

A variety of thermoplastic polymers, such as polypropylene (PP) and polyamide, are incorporated into the composition of these composites [56].

CAD/CAM technology has enabled the fabrication of custom-milled posts and cores, offering superior canal adaptation and mechanical performance compared to prefabricated fiber posts. Modern materials such as Trilor^®^ (fiber-reinforced epoxy resin) and PEKK (a high-performance polymer from the PAEK family) provide enhanced strength, elasticity closer to dentin, and versatile manufacturing options. Recent research has focused on improving their bonding characteristics and evaluating their fracture resistance under clinically relevant conditions, positioning them as promising alternatives for the future of post-endodontic restorations [61].

Recent advancements in regenerative endodontics have explored the use of polymer-based scaffolds to facilitate the biological repair and regeneration of dental pulp tissues. Natural polymers such as collagen and synthetic biodegradable polymers like polycaprolactone (PCL) have been widely investigated for their ability to support cellular adhesion, proliferation, and differentiation within the root canal space.

Regenerating dental pulp using tissue engineering technologies remains a major challenge in dentistry, with scaffold materials playing a crucial role. An ideal scaffold must be safe, biodegradable, biocompatible, and promote cell growth while possessing appropriate porosity, pore size, and interconnectivity to support tissue formation. Recent advances focus on natural and synthetic polymer scaffolds with favorable mechanical and biological properties, offering promising environments for cell activation and regeneration. When combined with stem cells and growth factors, these polymer scaffolds have shown strong potential to support and accelerate the revitalization of dental pulp tissue [62,63].

The review of Sugiaman et al. highlights the application of both natural and synthetic polymeric scaffolds in dental pulp tissue engineering. The authors emphasize that when combined with stem cells and growth factors, these scaffolds play a pivotal role in supporting cellular proliferation and differentiation necessary for pulp tissue regeneration.

Polymer-based scaffolds have become essential tools in regenerative dentistry by supporting the restoration of dental tissues through tissue engineering. Natural polymers like collagen and chitosan provide excellent biocompatibility, while synthetic polymers such as polylactic acid (PLA) and polycaprolactone lactide copolymer (PCL) offer controllable mechanical properties and degradation rates. Advances in bioactive molecule incorporation, nanotechnology, and hybrid scaffold development have enhanced both biological and mechanical performance. Innovations like 3D bioprinting and stimuli-responsive materials are further optimizing scaffold design, paving the way for more precise and effective regenerative treatments [64].

A summary of polymer based materials used in endodontic applications, including sealers and regenerative approaches, is provided in Table 4.

Clinical relevance: Polymeric sealers and resin-based obturation approaches aim to improve adaptation and sealing. Fiber reinforced composite posts provide a conservative option for restoring structurally compromised teeth when post selection matches root anatomy and adhesive steps are controlled [51,58].

Limitations and evidence gaps: Reported outcomes vary with operator technique and case selection, and surrogate endpoints such as microleakage and push out strength do not always predict long term survival. Robust clinical trials comparing contemporary sealers and post systems with standardized failure definitions remain limited [50,58].

### 3.5. Implantology

Polyetheretherketone (PEEK) and polyetherketoneketone (PEKK) have been increasingly utilized in implant dentistry due to their favorable mechanical properties, biocompatibility, and aesthetic advantages. These materials are employed in various components, including healing abutments, temporary abutments, and provisional crowns.

Advances in implantology increasingly focus on polymer-based materials like PEEK and PEKK, offering aesthetic, biologically compatible, and mechanically strong alternatives to traditional metal alloys. Unlike earlier thermoplastic resins, which lacked sufficient rigidity, PEEK and PEKK provide better support and longevity of temporary abutments, implants, and prosthetic components. Their bone-like elasticity and esthetic advantages make them highly attractive in modern dental treatments. Compared to zirconia, these polymers overcome limitations such as brittleness and low-temperature degradation, paving the way for next-generation implant solutions [65].

A clinical study demonstrated that PEEK, a high-performance polymer, is a viable material for provisional fixed dental prostheses (FDPs) during implant treatment. Fabricated using the lost wax technique and retained by reciprocated guide surfaces of hexagonal healing abutments, custom PEEK FDPs supported 166 implants across 20 patients. Over the treatment periods (approximately 7 months for maxillary and 2 months for mandibular cases), the PEEK frameworks showed no fractures, highlighting their excellent mechanical durability. Although prosthetic complications like veneer debonding and the need for supplementary screw retention were reported, all issues were managed intraorally. The findings support the use of PEEK for provisional restorations in both partially and fully edentulous cases, offering a strong, biocompatible, and adaptable alternative to traditional materials during the implant healing phase [19].

PEEK is increasingly used in implant-supported fixed dental prostheses (IFDPs) due to its cushioning effect, biocompatibility, light weight, and superior esthetics compared to metal frameworks. Studies show that PEEK frameworks offer sufficient fracture resistance and strong bonding with composite resins, allowing easier intraoral repairs. Although marginal adaptation can be slightly less precise than zirconia, PEEK provides significant advantages for patients with metal allergies or aesthetic demands. However, long-term clinical studies are still needed to fully validate its widespread application [66].

The biological response to PEEK-based dental implants is underexplored area. While PEEK is biocompatible, the long-term interaction of this material with the oral tissues has not been thoroughly examined. This is particularly important given the unique microbiological environment of the oral cavity, which could affect the stability and integrity of the implants. Future studies should focus on the microbial colonization of PEEK surfaces and the implications for periodontal health and systemic responses [67,68,69].

Research published in September 2024 explores the novel application of Polyether ether ketone (PEEK) in dental prosthetics, emphasizing its enhanced performance through innovative surface modifications and AI-driven optimization. Surface-treated PEEK exhibited up to a 30% increase in adhesion strength and demonstrated superior mechanical resilience, maintaining high tensile and compressive strength alongside improved fatigue resistance. Biocompatibility assessments confirmed PEEK’s non-cytotoxic nature and affirmed its safety for bone implant use. Moreover, lifecycle analysis indicated PEEK’s potential to reduce the environmental footprint of dental material production by lowering carbon emissions compared to traditional materials. AI-based predictive modeling further optimized material properties, ensuring more precise improvements in mechanical performance. Collectively, these findings position PEEK as a sustainable, high-performance alternative poised for broader clinical adoption in dentistry [70].

The use of 3D-printed surgical guides for implant placement has become common. These guides are typically printed from photopolymer resins (acrylate or epoxy-based) that are rigid and sterilizable. They precisely direct the drilling trajectory. This is an excellent example of a “diagnostic” polymer application overlapping implantology. Furthermore, other accessories like custom healing abutment shapers, radiographic stents (with embedded markers for imaging), etc., are often made of PMMA or resin.

Guided bone regeneration (GBR) is a key technique used to rebuild the alveolar bone in preparation for dental implant placement. Its biological foundation lies in providing a stable and immobile environment that supports bone growth by allowing the release of growth factors and preserving blood supply in areas of bone loss. The core principle of GBR is the use of a barrier membrane to prevent fast-growing soft tissue from invading the bone defect area, giving bone cells the necessary space to regenerate [71].

Membranes used in guided bone regeneration (GBR) must demonstrate high biocompatibility and long-term functional stability. Their primary role is to maintain the shape and mechanical integrity of the defect area while preventing unwanted cells and tissues from entering and protecting the developing bone tissue [12]. In addition, GBR membranes are categorized according to their composition and biological activity into bioabsorbable, non-resorbable, and those made from metals or inorganic compounds [72].

After the successful introduction of bioresorbable sutures for surgical wound closure, the idea of producing bone reconstruction and fixation devices from bioresorbable polymers emerged in the mid 1960s. Their use in treating facial fractures was first documented in 1971. Since then, numerous biomaterials have been developed and tested [73].

Bioabsorbable membranes are now regarded as the gold standard for lateral bone augmentation. Among them, those produced from natural native porcine collagen (NPCM) are the most widely used because of their excellent biocompatibility, ability to exclude undesirable cells, and ease of surgical handling [12]. However, these collagen membranes degrade rapidly during postoperative healing, losing about half of their thickness within the first two to four weeks and being fully resorbed within four to twelve weeks. In addition, NPCM membranes lack sufficient rigidity and tend to collapse even under mild mechanical pressure. To address these drawbacks, cross linked collagen membranes (CMs) have been introduced as an alternative. Cross linking improves their mechanical stability and resistance to degradation, allowing them to provide longer term support during tissue healing. However, this modification also increases the likelihood of postoperative complications, including foreign body reactions, delayed vascularization, inflammation, and reduced tissue integration, which can ultimately limit bone regeneration. In response, researchers have turned toward developing synthetic bioabsorbable barrier membranes using polymers such as poly lactic acid (PLA), poly glycolic acid (PGA), poly epsilon caprolactone (PCL), poly hydroxyl valeric acid, poly hydroxyl butyric acid, and their various copolymers [74].

Poly lactic acid (PLA) is a thermoplastic polymer produced through the fermentation of renewable sources such as corn starch, sugarcane, biomass, and other plant-based materials. It is now widely used across multiple industries, including food packaging, textiles, agriculture, and cosmetics [75,76,77].

Because of its favorable properties including biocompatibility, biodegradability, stiffness, non-toxicity, durability, and easy resorption, PLA is regarded as one of the most suitable biomaterials. However, its use is limited by low mechanical strength, poor heat resistance, and machining challenges. These drawbacks can be addressed through physical or chemical modifications. For instance, blending PLA with compatible copolymers can significantly enhance its structure, improving strength, toughness, and thermal stability [78].

Polyglycolic acid (PGA) is a highly crystalline polymer recognized for its strong mechanical properties and was one of the earliest uniform polymers explored for biomedical use. However, because it degrades rapidly, pure PGA has had limited application in maxillofacial surgery [31]. Although it possesses high tensile strength [32], its short degradation time does not allow sufficient bone healing. Furthermore, PGA degradation can trigger local inflammation, bone resorption, and sterile abscesses due to the production of acidic by-products [33,34]. Consequently, pure PGA is rarely used on its own and is often blended with other resorbable biomaterials. These combinations form copolymers that integrate the advantages of each constituent polymer while offering improved and more controllable degradation rates.

Second-generation bioresorbable materials primarily include copolymers of PGA, poly L lactic acid (PLLA), and poly D lactic acid (PDLA). Among these, the PLLA/PGA copolymer is the most promising, as its mechanical strength and rate of resorption can be finely adjusted by altering the proportion between PLLA and PGA [73].

Resorbable membranes are commonly used because they are hydrophilic and easy to handle, but they often lack the strength to maintain the proper space required for healing in larger bone defects or extraction sockets. To address this, non-resorbable polytetrafluoroethylene (PTFE) membranes were developed. Their main advantage is that they retain their shape and structural integrity during the healing process, offering both biocompatibility and rigidity for effective space maintenance. Additionally, PTFE membranes outperform resorbable ones in maintaining the space needed for bone regeneration and allow for less extensive flap reflection, which helps preserve keratinized gingival tissue [71].

In clinical settings, non-resorbable membranes are usually made of polytetrafluoroethylene (PTFE) and are available in two main forms—expanded (e PTFE) and dense (d PTFE)—with some versions reinforced with titanium. The d PTFE type features a smooth, fibril-free surface and very low porosity, which helps reduce bacterial attachment. Although limited bacterial migration along the internal surface has been observed, studies have shown that a high proportion of new bone formation is still achieved by the end of the guided bone regeneration process [79].

In implantology, polymers are thus not the headline (which is usually the titanium implant), but they play many supporting and some leading roles.

The main polymeric materials applied in implantology and guided bone regeneration, together with their key properties, are summarized in Table 5.

Clinical relevance: In implant dentistry, polymers are most clinically relevant as barrier membranes and scaffolds for guided tissue or guided bone regeneration, where handling, space maintenance, and resorption profile influence defect stability and soft tissue exclusion [71,72,80].

Limitations and evidence gaps: Membrane exposure, infection risk, and mismatch between resorption and healing timelines remain major failure drivers. Evidence for newer polymer composites and drug loaded scaffolds is still emerging and heterogeneous [71,72].

### 3.6. Polymers in the Field of Impression Materials

In addition to the main clinical disciplines, polymers contribute to diagnostic procedures and emerging high-tech applications in dentistry. “Diagnostics” here refers to materials and devices used to obtain clinical information (like impressions or imaging guides), as well as new tools for disease detection. Meanwhile, emerging technologies span innovations like 3D printing, smart materials, and sustainable (“green”) dentistry solutions that cut across multiple fields.

Making accurate impressions of teeth and oral tissues is a foundational diagnostic step in dentistry. Nearly all dental impression materials are based on polymer chemistry. Modern dental impression materials are designed to provide accurate detail reproduction and are typically well accepted by patients due to their ease of use and comfort. Despite these advantages, their polymer-based nature introduces certain limitations, particularly concerning dimensional stability over time. Factors such as polymerization shrinkage, moisture absorption, and thermal sensitivity can compromise the precision of the impression if not properly managed, which remains a critical consideration in restorative and prosthetic procedures [81].

Impression materials in dentistry must ensure accuracy, ease of handling, patient comfort, and resistance to tearing upon removal. Among these, hydrocolloid materials—particularly alginate—are widely used due to their affordability, hydrophilicity, biocompatibility, and straightforward manipulation, making them ideal for diagnostic impressions in routine clinical practice [82,83,84].

Dental alginate has relatively low tear strength (0.4–0.7 N/mm), making it more prone to tearing compared to elastomeric impression materials like polysulfide, which ranges from 2.5 to 7.0 N/mm. To enhance its tear resistance, incorporating fillers into the alginate formulation is a commonly explored strategy [82,85,86,87].

While both inorganic and organic fillers are used, organic fillers are preferred due to their superior biocompatibility, lower density, cost-effectiveness, recyclability, non-toxicity, and ease of processing. A common example is methyl methacrylate (MMA)-based fillers [88,89].

Polymethyl methacrylate (PMMA) is commonly reinforced with fillers to improve its flexural strength and modulus for denture base applications. However, its use to enhance alginate impression materials has not been explored. Research by Acosta et al. suggests that MMA, the monomer of PMMA, can enhance dimensional stability, density, and mechanical properties when polymerized into other materials [89,90].

In their study, Ignatius Enrico Paskatrianto, Veni Takarini, and Kosterman Usri aimed to assess the changes in tear strength of alginate impression materials following reinforcement with polymethyl methacrylate (PMMA) as an organic filler. As a result of this study, the incorporation of PMMA fillers into alginate led to an improvement in the material’s tear strength. Variations in PMMA filler concentration produced differing effects, with the most significant enhancement observed at a concentration of 5 wt%. Zafar noted that polymerized MMA can increase material toxicity, with self-curing variants being more toxic than heat-curing ones. In this study, no initiator was used, meaning MMA did not chemically bond with alginate, and thus no increase in toxicity was observed. Given alginate’s brief intraoral use (about one minute) and MMA’s longer polymerization time (~10 min), the risk of toxicity remains minimal. However, further research is recommended on the biocompatibility of PMMA fillers, particularly those that may chemically interact with alginate. The use of heat-curing PMMA fillers is advisable due to their lower toxicity profile [9,91].

Elastomeric dental impression materials are widely used for capturing highly accurate molds of teeth and oral tissues in prosthodontics. They are classified as non-aqueous elastomers (as opposed to hydrocolloid alginate) and include polysulfides, polyethers, and addition silicones (polyvinyl siloxanes, PVS) [92].

Polysulfides were the first elastomeric impression materials (developed in the 1950s). They are supplied as a two-paste system (base and catalyst). The base paste contains a polysulfide prepolymer (a mercaptan-terminated polymer), along with fillers and plasticizer, while the catalyst contains an oxidizing agent (usually lead(II) oxide), plus inert oils and sulfur to promote curing. When mixed, condensation polymerization occurs: the mercaptan (-SH) groups of the prepolymer react with lead oxide to form a cross-linked polysulfide rubber, releasing water as a byproduct. Because water is produced and can evaporate, immediate pouring of the gypsum cast is required to avoid distortion. Polysulfide was long favored for its extended working time and tolerance for a longer setting time, which can be useful for taking multiple impressions or full-arch impressions. However, the setting reaction’s byproduct leads to poor long-term dimensional stability, so these impressions cannot be stored for long. Polysulfide materials typically have a noticeable brown color (from lead oxide) and a sulfur odor and taste that many patients find unpleasant. Due to these drawbacks and messy handling, polysulfides are now rarely used except in complete denture cases, having been largely supplanted by silicones and polyethers.

Despite their issues, polysulfides possess some advantageous properties. They are the least rigid (most flexible) of the elastomers, which means a set polysulfide impression can be easily removed from undercut areas without tearing. In fact, polysulfide has excellent tear strength, minimizing the risk of the impression material tearing even in thin sections or around sharp edges. Its flow prior setting is also very good, allowing excellent detail reproduction of the oral tissues. Polysulfide was traditionally considered moderately tolerant of moisture (some texts even label it “hydrophilic”), and indeed its wettability is somewhat better than unmodified PVS. However, it is still generally a hydrophobic, rubber-based material that cannot displace water as effectively as true hydrophilic materials. Clinically, a well-made polysulfide impression yields accurate casts if poured promptly, but any delay or improper storage causes significant dimensional change. Moreover, the long intraoral set time (up to ~10 min) and bad odor/taste reduce patient comfort and make polysulfide less convenient in practice. For these reasons, polysulfide has largely been replaced by newer elastomers with better dimensional stability and patient acceptability [92].

Despite being introduced in the 1960s and 1970s, polyether impression materials remain widely used, making them one of the longest-standing modern elastomers in dentistry. They consist of a base paste with polyether prepolymers and a catalyst containing sulfonate esters. The setting mechanism is a cationic ring-opening polymerization, which produces a rigid, crosslinked rubber without releasing volatile byproducts—ensuring excellent dimensional stability and accuracy, even after disinfection or delayed pouring.

Polyethers are intrinsically hydrophilic, enabling excellent wettability and precise detail capture in moist environments—superior to hydrophobic materials like unmodified Polyvinyl Siloxane (PVS). This makes them especially effective for impressions involving saliva or blood. Additionally, they exhibit excellent elastic recovery and high tensile strength, though their high stiffness can complicate removal from undercut areas. To address this, newer formulations have been made slightly more flexible.

Their main clinical advantages include high accuracy, moisture tolerance, and reliable gypsum compatibility. Limitations include a short working time, moderate tear resistance, and a tendency to absorb water if overexposed during disinfection. While more expensive than some alternatives and slightly bitter in taste, polyether remains a preferred choice for complex cases and moist environments, especially when handled carefully to manage its rigidity [92,93,94,95,96,97].

Polyvinyl siloxanes (PVS)—also known as addition silicones—are widely recognized as the gold standard for fixed prosthodontic impressions due to their excellent dimensional stability, elastic recovery, and fine detail reproduction. Introduced in the 1970s, PVS materials come as a two-part system (base and catalyst) in various viscosities. Their setting reaction involves no volatile byproducts, which ensures minimal polymerization shrinkage (<0.2%) and allows impressions to be poured multiple times or stored for extended periods with negligible distortion.

PVS offers rapid setting times (2–6 min), 99% elastic recovery, and high tear resistance, especially in modern reinforced versions. These properties enable easy removal from the mouth while preserving accuracy. However, traditional PVS is hydrophobic, potentially trapping moisture and compromising detail in wet fields. To overcome this, manufacturers have developed hydrophilized versions by adding surfactants, which improve wettability and allow reliable performance under moist conditions—though polyether materials still outperform PVS in inherently wet environments.

Clinically, PVS is odorless, tasteless, and less stiff than polyether, enhancing patient comfort. It is sensitive to sulfur contamination (e.g., from latex gloves), which can inhibit polymerization; hence, vinyl gloves are recommended. Overall, PVS combines precision, dimensional accuracy, and ease of handling, making it ideal for crowns, bridges, and implant impressions—a material of choice across restorative disciplines [92,93,97,98,99].

Condensation silicone is one of the most economical elastomeric impression materials used in dentistry. While it provides acceptable accuracy and ease of manipulation, its main limitation lies in its tendency to shrink after setting. Because of this dimensional instability, impressions made with condensation silicone must be poured immediately to ensure accuracy.

This material is available in several consistencies, including extra low, low, medium, and putty forms, allowing clinicians to choose the most appropriate viscosity for different clinical situations. It is supplied in various packaging forms: collapsible tubes for the base and catalyst in the medium body version, jars for the putty form, and syringes for the ultra-low and low viscosity types.

In terms of composition, the base contains hydroxy terminated polydimethylsiloxane, a polymer combined with fillers to adjust viscosity. The accelerator consists of tetraethyl silicate and tin octoate, which acts as a catalyst. The material is set through a condensation polymerization reaction that produces alcohol as a byproduct. This alcohol evaporates during the reaction, leading to the slight contraction of the material.

Condensation silicones have several advantages, including good elastic recovery, a pleasant odor and taste, and the ability to reproduce fine surface details accurately. However, their disadvantages are significant. They are hydrophobic, prone to dimensional changes over time, and their long-term stability is compromised by the release of volatile byproducts during the setting process. Consequently, while condensation silicone remains a cost-effective choice, its clinical use requires careful handling and immediate model pouring to maintain precision [92].

In recent years, material scientists have introduced hybrid impression materials that combine polyether and PVS chemistries to leverage the strengths of each. These products are often called vinyl polyether silicone (VPES) or polyvinyl siloxane ether (PVES). Chemically, a VPES material is created by grafting polyether segments onto a vinyl-polysiloxane network or otherwise combining the two polymer systems. The goal is to achieve instant hydrophilicity (from the polyether component) together with the improved elasticity and stability of additional silicone. One manufacturer description is that the polyether part provides inherent wettability and flow, while the siloxane part provides strength, elasticity, and low shrinkage. These hybrid materials have been described as “pioneering” elastomers that meet advanced clinical demands.

Recent studies (2020–2024) have evaluated whether the hybrid materials truly realize better performance. Overall, the findings are promising: a 2024 systematic review and meta-analysis compiled data from various studies and found that vinyl polyether silicone (VPES) indeed showed improved hydrophilicity and tensile strength compared to both polyether and PVS alone. The hydrophilicity, in particular, was significantly enhanced—the polyether component clearly imparts a low contact angle, which can improve detail reproduction in moist environments. At the same time, the review found no statistically significant differences in dimensional accuracy among VPES, PVS, and polyether—indicating that the hybrid maintained accuracy on par with the excellent standards of PVS and polyether [97,100].

Another study using digital analysis noted the hybrid had intermediate dimensional accuracy: slightly better than polyether, though not quite as exact as the best PVS in a specific test.

In terms of elastic recovery and tear strength, hybrids tend to combine the merits: one comparative study reported the new hybrid had high elastic recovery and tear resistance, similar to PVS, and markedly better than an older polyether [93].

Essentially, hybrid VPES materials inherit the polyether’s ability to wet and flow, and the PVS’s ability to rebound and remain dimensionally stable. Clinically, this means easier impressions in moist conditions without needing surfactants, and reliable removal with low deformation. Researchers have noted that further optimization is needed to perfect the compatibility of the two chemistries (some early VPES versions had issues like shorter working time or technique sensitivity) [97].

Despite the rapid progress of digital technologies and the growing adoption of intraoral scanners in modern dentistry, conventional impression materials continue to hold an essential role in clinical practice and cannot yet be completely replaced [101].

### 3.7. Digital Impressions and Intraoral Scanning Workflows

Digital impressions replace the conventional polymer-based impression with an optical capture of the intraoral geometry. In practice, this shifts the clinical workflow from selecting an impression material and tray system to managing scan strategy, soft tissue control, and surface conditions such as saliva, reflections, and subgingival margins. From a polymer’s perspective, the role of classic elastomeric materials becomes more selective, while polymeric components remain relevant through scan body materials, temporary restorations, and printable resins used for models, splints, and surgical guides in downstream CAD CAM workflows [101,102].

Clinically, intraoral scanning is often advantageous when patient comfort, speed, and digital integration are priorities, especially for single unit restorations and short span cases where the scanned area is limited. However, accuracy and reliability can become more challenging as the scanned area increases, because cumulative stitching errors, mobile soft tissues, and limited access can degrade the fidelity of a full-arch record. In addition, deep or bleeding margins can reduce optical data quality, where hydrophilic conventional materials may still provide more predictable detail capture [101,102,103].

In implant impressions, the digital workflow relies on scan bodies that are commonly manufactured from polymers such as PEEK or resin-based composites. The dimensional stability of the scan body and the precision of its seating on the implant interface become critical, because any rotational or axial discrepancy propagates directly into the virtual implant position. For complex full-arch implant cases, clinicians may still prefer conventional impression approaches when optical capture is compromised by limited interarch space, soft tissue collapse, or the need for controlled splinting concepts [104].

A practical decision point is therefore case selection. Digital impressions may be favored for patients with gag reflex, time constraints, and workflows that benefit from rapid iteration and electronic transfer. Conventional elastomeric impressions remain valuable for challenging margin conditions, extensive edentulous spans, and situations where moisture control is limited. As scanner hardware, reconstruction algorithms, and chairside manufacturing continue to mature, digital impressions are likely to further reduce the routine use of conventional materials, but they do not fully eliminate the need for polymer-based impression materials in demanding clinical scenarios [101,105].

Manufacturers continue to refine VPES formulations, and these materials are increasingly being adopted for cases where a clinician desires the handling of a polyether with the stability of a silicone.

Elastomeric impression materials have evolved significantly, and each type’s polymeric structure underpins its clinical behavior. By understanding the chemistry (cross-linking mechanism and composition) and performance profile of each material, clinicians can select the appropriate impression material to ensure an accurate impression and a well-fitting final restoration.

A comparative overview of polymer based impression materials and their relevant properties is presented in Table 6.

## 4. Discussion

In the domain of prosthodontics, the reviewed literature indicates that polymeric materials are deeply integrated into nearly all aspects of tooth replacement and oral rehabilitation. Conventional polymethyl methacrylate (PMMA) remains a cornerstone for fabricating dentures (bases and artificial teeth), obturators, custom trays, provisional crowns, and even occlusal splints. Its popularity is due to favorable properties such as low density, affordability, ease of processing, and acceptable aesthetics. Recent advances have focused on enhancing PMMA’s mechanical performance—for example, fiber-reinforced and nanoparticle-modified denture acrylics—to improve strength, reduce water sorption, and increase longevity [9].

Another key finding is the emergence of high-performance polymers like polyetheretherketone (PEEK) as metal-free alternatives for frameworks in removable and fixed prostheses. PEEK has been applied in crowns, fixed partial dentures, and post-and-core systems, offering a modulus of elasticity closer to bone than metal alloys and thereby distributing occlusal stresses more evenly. Clinically, this means polymer frameworks (e.g., PEEK or carbon-fiber reinforced resins) can reduce stress on abutment teeth and potentially minimize prosthesis-related bone resorption or root fractures. An important implication is that these materials enable metal-free prosthodontics, benefiting patients with metal allergies or esthetic concerns. However, the review also highlighted that PEEK’s chemically inert surface poses bonding challenges, necessitating specialized surface treatments (abrasion, etching, or primers) to achieve reliable adhesive luting. From a clinical standpoint, dentists incorporating PEEK frameworks must adopt these pretreatment protocols to ensure durable bond strength. Overall, the prosthodontic use of polymers—from traditional denture resins to modern high-performance polymers—has broadened treatment options, improved patient comfort (lighter, more esthetic prostheses), and reduced biological side-effects (no metal ion release and good biocompatibility) [13].

Yet, practitioners should remain mindful of technique sensitivities (such as proper bonding of inert polymers) and the still-limited long-term clinical data on newer materials [107].

The review underscored that restorative dentistry has been revolutionized by polymer-based materials, with resin composites and adhesives taking precedence over traditional amalgams and cements in many applications. Resin-based composites (RBCs) are now ubiquitous for direct restorations in anterior and posterior teeth, as well as for pit-and-fissure sealants, core buildups, inlays, onlays, and even crowns.

Contemporary RBC formulations consist of a polymerizable organic matrix (commonly methacrylate resins) heavily loaded with inorganic fillers and silane coupling agents. A key finding is that significant improvements have been achieved by nanotechnology—the incorporation of nanofillers has markedly enhanced composite performance, yielding higher strength, smoother polished surfaces, and improved esthetics (gloss and color stability) while also reducing biofilm adhesion [108].

Clinically, these advancements lead to production of more durable and lifelike restorations that can better withstand masticatory forces and resist staining or wear overtime. Similarly, dental adhesives (bonding agents), which are typically polymeric resin systems, have evolved through multiple generations to enable dependable bonding of restorations to enamel and dentin. The review notes that universal adhesives containing functional monomers (e.g., 10-methacryloyloxydecyl dihydrogen phosphate) now achieve strong, durable bonds to both tooth structure and various restorative materials under simplified application protocols. The clinical implication is that minimal invasive dentistry is bolstered by reliable adhesive techniques—dentists can preserve more tooth structure and still achieve high retention and seal of restorations. Furthermore, polymer-based glass-ionomer and resin-modified cements remain important for certain restorations and luting purposes, offering fluoride release and chemical bonding in low-load areas [109].

Overall, the restorative domain’s key takeaway is that polymeric materials (composites and bonding systems) have enabled superior aesthetics and function in tooth-colored restorations, though the review emphasizes the need for continued improvements in properties like polymerization shrinkage stress and wear resistance. From an evidence-based practice perspective, clinicians should be aware that while current resin composites are state-of-the-art, factors such as proper curing technique and adhesive application greatly influence clinical outcomes; this narrative synthesis reinforces that technique sensitivity and operator skill remain critical alongside material advances.

In orthodontics, the review identified polymers as central to both traditional and modern treatment modalities. Even in conventional fixed braces, polymers find use as esthetic bracket alternatives (plastic or composite brackets) and in elastomeric ligatures and power chains. While metal brackets still dominate for strength, polymer-based brackets offer superior aesthetics but may have higher friction and wear—an important clinical trade-off noted in the literature [109].

The most transformative development in this specialty, however, is the rise in clear aligner therapy, which is fundamentally enabled by advanced thermoplastic polymers. The review highlights that clear aligners (exemplified by Invisalign and similar systems) are typically fabricated from medical-grade thermoplastic polymers such as polyurethanes, polyesters (PETG), and polypropylenes, either individually or in proprietary blends. These materials provide the required combination of strength, flexibility, and transparency. Key findings show that material composition strongly influences aligner performance—differences in polymer formulation can affect force delivery, aligner stiffness, and patient comfort. Clinically, the implications of polymeric aligners are profound: they offer an aesthetic and comfortable alternative to fixed braces, improving patient acceptance and oral hygiene maintenance (since aligners are removable during cleaning). Patients, especially adults, experience fewer dietary restrictions and periodontal side-effects with aligners, as noted by the review’s assessment of periodontal health outcomes [110,111].

That said, the discussion also points out limitations—thermoplastic aligners can exhibit loss of force overtime due to material fatigue and intraoral conditions (thermal and mechanical stresses), meaning they must be engineered and scheduled appropriately to achieve effective tooth movement. The reviewed literature suggests that ongoing material research (e.g., incorporating shape-memory polymers or multi-layered aligner foils) is aimed at overcoming these issues. In summary, polymeric materials have enabled a paradigm shift in orthodontics toward nearly invisible and patient-friendly appliances, though achieving biomechanical predictability with polymers requires careful material selection and treatment planning.

Polymers are essential in endodontics for both root canal obturation and post-treatment tooth restoration. The review highlights that gutta-percha, a natural rubber polymer (trans-1,4-polyisoprene), remains the gold-standard material for root canal filling due to its biocompatibility, dimensional stability, reversibility in retreatment and remains reliable for long-term success [51].

While alternatives like Resilon (a polycaprolactone-based system) aimed to form monoblock seals for improved outcomes, clinical studies have not demonstrated clear clinical advantages, so gutta-percha continues to dominate [52].

Polymeric sealers, especially epoxy and methacrylate-based formulations, are used to enhance sealing and bonding inside canals.

Fiber-reinforced composite posts (glass or carbon fiber in resin) and PEEK posts are important for reinforcing endodontically treated teeth. These materials have an elastic modulus close to dentin, allowing for better stress distribution and significantly lower risk of root fractures compared to metal posts [13]. The implication for practice is significant: the shift from metal to fiber/polymer posts in post-endodontic restorations has improved the long-term prognosis of structurally compromised teeth by preserving root integrity. Attention is also drawn to regenerative endodontics as an emerging subdomain—here, biodegradable polymers (such as polylactic acid and polyglycolic acid scaffolds) have been investigated for delivering stem cells or growth factors to regenerate pulp–dentin complex. While these tissue-engineering approaches are in early experimental stages, they underscore a forward-looking role of polymers in endodontics beyond traditional filling materials. In conclusion, polymeric materials in endodontics have both well-established and novel applications: they are instrumental for effective debridement sealing and restoration today, and they hold promises for biologically based therapies in the future. Clinicians should continue to rely on the proven polymer materials (like gutta-percha and fiber posts) while keeping an eye on developing evidence for regenerative uses.

The review delineated several critical applications of polymers in implant dentistry, highlighting how polymers complement the predominantly metallic nature of implants. One major finding is the use of high-performance polymer frameworks and abutments as lightweight, metal-free alternatives in implant-supported prostheses. Materials such as PEEK and its kin (PEKK) have been explored for custom implant abutments, interim implant restorations, and even experimental one-piece implants. These polymers exhibit excellent biocompatibility and radiolucency and possess an elasticity modulus closer to bone, which may help in mitigating stress shielding around implants. Clinically, PEEK provisional abutments and frameworks can improve patient comfort (by reducing metal taste or allergies) and make postoperative imaging easier due to lack of radiographic scatter. However, as with their use in tooth-borne prosthetics, a noted challenge is ensuring durable bonding to veneering resins or cements given PEEK’s inert surface [13].

The most impactful polymeric contribution in implantology, as emphasized by this review, is in guided tissue regeneration for implant site development. Synthetic polymer membranes (e.g., made of polylactide, polyglactin, or expanded PTFE) are widely used as barrier membranes in guided bone regeneration procedures. These membranes play a fundamental role in separating bone defects from soft tissue, thereby creating a secluded space for bone regrowth prior to or simultaneous with implant placement. The review concludes that polymeric barrier membranes significantly improve the success of implant treatments by facilitating bone healing and defect fill, with the added advantage that bioresorbable polymer membranes obviate the need for a second surgery to remove them [80].

Additionally, polymer-based scaffolds and hydrogels are being investigated for delivering growth factors or cells to enhance osseointegration and bone regeneration around implants. From a clinical perspective, the current implication is clear: polymers have enabled more predictable bone augmentation and implant restoration protocols. Membranes and scaffolds offer surgeons versatile tools to manage challenging implant cases (such as deficient ridges), though proper case selection and handling of these materials (e.g., ensuring membrane stability and hydration) are crucial for success. In summary, polymeric materials support implantology by improving surgical outcomes and providing new prosthetic options, and ongoing research is rapidly expanding their utility in this domain.

The evolution of dental impression materials has been a gradual process spanning several centuries, shaped by the pursuit of precision, stability, and patient comfort. The earliest recorded dental impression dates back to the seventeenth century when the German surgeon Gottfried Purmann used wax to replicate jaw and tooth structures. In the eighteenth century, Philip Pfaff advanced the technique by using wax impressions of edentulous jaws and creating plaster casts, laying the foundation for modern prosthodontics. Over time, materials such as zinc oxide eugenol and compound were introduced but were limited by their inability to accurately reproduce undercuts without distortion or fracture. The introduction of reversible hydrocolloids in 1925 and irreversible hydrocolloids in 1941 marked a significant step forward, although these materials suffered from shrinkage caused by water loss. The mid-twentieth century brought major innovations with the development of elastomeric materials like polysulfide, polyether, and polyvinyl siloxane (PVS), which provided improved accuracy and dimensional stability, revolutionizing dental impressions.

Among the commonly used materials, alginate—an irreversible hydrocolloid—remains popular for its affordability, ease of use, and hydrophilic properties that allow accurate replication of moist oral tissues. However, its dimensional instability caused by water absorption and loss limits its long-term use, restricting it mainly to diagnostic and temporary models. Polyether, introduced in the 1960s, offered superior wettability and precision in moist environments. Its cationic polymerization reaction produces no byproducts, ensuring excellent accuracy, though its water absorption makes it unsuitable for humid storage. Polyvinyl siloxane (PVS), introduced in the 1970s, soon became the material of choice due to its outstanding dimensional stability, elasticity, and ability to reproduce fine details with minimal distortion. It sets through an addition reaction with no volatile byproducts, but the release of small amounts of hydrogen gas requires a waiting period before pouring to avoid defects. PVS is also sensitive to sulfur-containing materials like latex, which can inhibit polymerization, demanding careful clinical handling.

Further innovation led to the creation of hybrid materials like vinyl siloxanether, introduced in 2009, which combines the hydrophilic properties of polyether with the elasticity and accuracy of PVS. These materials are especially useful in moist clinical conditions such as narrow gingival crevices, though long-term studies on their accuracy remain limited. To improve PVS further, manufacturers introduced hydrophilic formulations containing surfactants, allowing better performance in humid conditions. Despite this, they remain hydrophobic before setting, sometimes resulting in inconsistent detail capture if excessive moisture is present.

Modern developments have also focused on patient comfort and clinical efficiency. Fast-set elastomeric materials were introduced to shorten chair time and assist patients with strong gag reflexes. Studies show that fast-setting polyether provides highly accurate impressions and excellent detail reproduction, while newer formulations like thiol-ene functionalized siloxane demonstrate rapid polymerization and fine surface capture. Additionally, alginate substitute materials emerged in the 1980s as cost-effective polyvinyl siloxane alternatives with superior mechanical properties, tear strength, and dimensional stability compared to traditional alginate. However, these materials must be poured immediately and do not allow for repeat impressions.

In summary, the history of impression materials reflects a continuous evolution from rudimentary wax molds to sophisticated elastomeric polymers. Each generation of materials has built upon the shortcomings of its predecessors, advancing toward greater accuracy, speed, and comfort. Modern impression materials like polyether, polyvinyl siloxane, and their hybrids now provide clinicians with reliable tools to produce precise dental restorations, embodying the intersection of chemistry, engineering, and clinical artistry in contemporary dentistry [106,112].

Clinical relevance: For conventional impressions, polyvinyl siloxane and polyether remain high accuracy standards, while digital impressions can improve workflow efficiency and patient comfort for many indications. Selection should be based on margin location, moisture control, and the need for full-arch accuracy [104,105].

Limitations and evidence gaps: Scanner accuracy can degrade in subgingival margins, reflective surfaces, and long span full-arch cases, and conventional materials remain necessary in specific scenarios. Comparative studies vary in devices, software versions, and reference standards, limiting generalizability [101,105].

## 5. Conclusions

Polymers have become indispensable in contemporary dentistry, shaping virtually every specialty through their unmatched versatility, biocompatibility, and adaptability. From prosthodontics to restorative dentistry, orthodontics, endodontics, and implantology, polymeric materials provide functional and aesthetic solutions that no other material class can replicate. Their tunable chemical structures allow for customization across a spectrum of rigidity, flexibility, transparency, and biodegradability, enabling precise alignment with clinical needs.

In conclusion, polymers occupy an irreplaceable role in modern dentistry, acting as the foundation of restorative, prosthetic, orthodontic, endodontic, and implant innovations. Their continuous evolution not only addresses clinical challenges but also expands the boundaries of what dental materials can achieve. The shift toward bioactive, sustainable, and digitally manufactured polymers ensures that dentistry remains aligned with both patient needs and environmental responsibility. As the field progresses, polymers will continue to serve not merely as substitutes for natural tissues but as intelligent, multifunctional materials that define the next generation of dental care.

## Figures and Tables

**Table 2:** Polymers in Restorative Dentistry.

Material	Composition/Type	Applications	Key Properties & Performance	Recent Innovations
Composite resin (universal hybrid)	Bis-GMA/UDMA/TEGDMA resin + fillers (glass, silica, zirconia)—light-cured	Direct anterior/posterior restorations, core buildups	Compressive strength ~300–400 MPa; wear rate similar to enamel for nanohybrids; polymerization shrinkage ~2% Good esthetics and polish [21,22].	Nanofillers for better wear and gloss; low-shrink monomers; bulk-fill technology for 4–5 mm curing; experimental antimicrobial and remineralizing additives [21,22]
Flowable composite	Lower filler content resin composite (light-cured)	Cavity liners, minimal restorations, pit/fissure sealants	Lower viscosity, adapts well but ~20–30% less strength than paste composite. Higher polymerization shrinkage due to less filler [21,22].	Bioactive flowables with Ca–P glass or ACP for remineralization; injectable resin ionomers for liners.
Resin-modified Glass Ionomer (RMGI)	Hybrid of acrylic monomers and glass-ionomer components (acidic resin + fluoroaluminosilicate glass)	Luting cement, liners, cervical lesions in high caries risk patients	Fluoride release over time; chemical bond to tooth; lower strength than composite (compressive ~150–200 MPa). Less sensitivity to moisture than pure GI [22].	New RMGIs with nano-hydroxyapatite to boost remineralization; bulk-fill RMGIs with higher depth of cure.
CAD/CAM composite or PICN blocks	Highly filled (>80 wt%) composite or polymer-ceramic interpenetrating network—industrially cured [29,32,33]	Milled inlays, onlays, crowns, implant veneers	Elastic modulus ~12–20 GPa (lower than ceramic ~65 GPa, more forgiving to opposing teeth); easier to mill (no firing needed). Good fracture toughness but can wear faster [29,32,33].	Introduction of fiber-reinforced CAD/CAM blocks; improvements in thermal cure to eliminate residual monomer for better stability; some blocks with bioactive fillers for ion release.
Sealant (unfilled resin)	Light-cure or self-cure Bis-GMA/TEGDMA resin (often translucent)	Pit and fissure sealant for caries prevention in molars/premolars	Low viscosity, penetrates pits. Bonds to enamel via tags. Wears down slowly over years; can be opaque or clear.	Addition of fluoride or calcium-releasing fillers; self-etching sealants that do not require separate etch step; colored sealants that fade to clear as an indicator for placement accuracy

Sources for numerical values in table: [21,22,29,32,33,34,35,36].

**Table 4:** Summarizes endodontic polymer-based materials.

Material	Endodontic Use	Composition/Type	Properties & Outcomes
Gutta-percha (conventional)	Core filling of root canals (with sealer)	Natural rubber polymer (trans-polyisoprene) with zinc oxide and radiopacifiers	Thermoplastic, softens ~60 °C. Inert when set, slight expansion on solvent exposure. Long history of success; does not bond to dentin—relies on sealer [58].
Resilon (polyester obturator)	Core filling (alternative to gutta-percha)—now mostly discontinued	Synthetic aliphatic polyester (polycaprolactone) with bioactive glass filler	Melt-point ~55 °C, can bond with resin sealer to dentin forming monoblock. Showed higher biodegradation and similar leakage as GP in studies; no longer widely used [51,58].
Epoxy resin sealer (e.g., AH Plus)	Root canal sealer	Two-part epoxy-based sealer (bisphenol-A epoxy + amine hardener)	Working time ~4 h, sets in 8 h into crosslinked solid. Very low solubility (<1%). Excellent long-term sealing, but no inherent antimicrobial activity. Gold standard sealer for many practitioners [53].
Methacrylate resin sealer (e.g., EndoREZ)	Root canal sealer (with bonding)	Urethane dimethacrylate-based resin sealer, light or dual cured	Can polymerize through canal if light reaches or via chemical cure. Bonds to dentin if dentin adhesive is applied. Shrinkage on cure can lead to gaps. Not as commonly used due to technique sensitivity.
Silicone sealer (e.g., GuttaFlow)	Root canal sealer	Polydimethylsiloxane (addition-cure silicone) with gutta-percha powder and silver particles	Sets with slight expansion (~0.2%), providing a tight seal. Very flowable and easy to use. Biocompatible. Some variants eliminate need for separate GP cone (acts as sealer + core) [58].
Fiber post (glass or carbon fiber)	Intraradicular post for core build-up in endodontically treated tooth	Bundles of glass or carbon fibers in epoxy resin matrix (prefabricated post)	Modulus closer to dentin, resulting in fewer root fractures. Bonds to resin cement. Allows light transmission if glass fiber (improves cement cure). Success rates high (~90 + % at 5 years in many studies) with mostly restorable failures (post debonding) rather than root break [58,61].
Fiber-reinforced composite (FRC) post-and-core systems	One-piece post and core made from fiber composite, or short fiber composite core materials	e.g., EverX composite core (short glass fibers in resin) used inside canal and crown as post-less build-up	FRC build-ups distribute stress, avoid stress concentration of a discrete post. In vitro shows high fracture resistance, with failures usually being core fracture rather than root. Still need more clinical data.
Collagen or PCL scaffold (experimental)	Regenerative endodontics scaffold for tissue engineering	Natural collagen sponge or synthetic polycaprolactone 3D scaffold placed in canal [62,63,64]	Provides 3D support for cell ingrowth in revitalization procedures. Biodegradable over time. Early case reports show potential for aiding pulp-like tissue formation, but not standard of care yet [62,63,64].

Sources for numerical values in table: [51,53,55,58,61,62,63,64].

**Table 5:** Summary for implantology.

Polymer/Item	Implantology Application	Properties/Role	Clinical Notes
PEEK (polyetheretherketone)	Healing caps, temporary abutments; frameworks for implant prostheses; experimental implant bodies	Rigid, strong polymer with bone-like modulus (~4 GPa) Highly biocompatible (inert) [19,65,66].	Reduces stress shielding when used as abutment; no metallic taste or color. As implant material, needs surface modification to osseointegrate (coatings or porosity). Good clinical success in provisional use; long-term implant usage under study.
PMMA (acrylic) and composite resins	Provisional implant crowns/bridges; interim dentures on implants; denture teeth in hybrids	PMMA: ease of fabrication, adequate strength short-term. Composite: better wear resistance, more esthetic for teeth.	Interim restorations protect implants during healing. In full-arch hybrids, acrylic/composite teeth act as shock absorbers vs. ceramic—but need periodic maintenance (teeth wear or fracture every few years).
ePTFE (Teflon)	Non-resorbable GBR membrane	Chemically inert, microporous PTFE membrane. Does not resorb, requires removal.	Excellent barrier for GBR; risk of infection if exposed due to porosity (bacteria can colonize). Dense PTFE membranes address that by eliminating pores, allowing even exposure without infection risk, at the expense of no nutrient diffusion.
PLA/PGA (bioresorbable polymers)	Resorbable GBR membranes; resorbable fixation screws or tacks for membranes	Biodegradable polyesters that hydrolyze to metabolic acids. Membranes typically maintain strength ~2–4 months before resorption [73].	Avoid second surgery for removal. New composites (PLA + bioactive glass) enhance osteogenesis. Sometimes used to make resorbable pins to secure membranes or bone graft materials; those pins slowly dissolve after bone heals.
Collagen (natural polymer)	Resorbable membranes; collagen plugs for sockets	Type I collagen from tendon or dermis, often cross-linked to last ~3–6 months in vivo [71,72].	Excellent tissue integration, very biocompatible Acts as scaffold for bone cells. Collagen plugs can aid initial clot stabilization in extraction sockets. Cross-linked versions resorb slower but may elicit more inflammation.
3D-printable resin (acrylate photopolymer)	Surgical implant guides; custom trays; custom healing abutments	Photocurable methacrylate-based polymer, rigid and accurate. Typically biocompatible resin class I (for mucosal contact <24 h) for guides [43].	Enables precise guided surgery. Must be sterilized (usually via cold soak, some autoclavable resins exist). Custom healing abutments can shape gum profile optimally—printed in biocompatible resin and cemented or attached to implants temporarily.

Sources for numerical values in table: [9,19,43,65,66,71,72,73].

**Table 6:** Summary for Polymers in the field of impression materials.

Polymer/Material	Application	Properties/Role	Clinical Notes
Alginate (Hydrocolloid)	Diagnostic impressions for study models, opposing casts, and orthodontic records	Hydrophilic, biocompatible, cost-effective, easy to mix and remove	Low tear strength (0.4–0.7 N/mm), poor dimensional stability, must be poured immediately; enhanced tear strength possible by adding fillers like PMMA or MMA [83,87].
Polymethyl Methacrylate (PMMA) (used as filler)	Reinforcement of alginate impression materials	Improves tear strength, density, and mechanical properties	Optimal reinforcement at ~5 wt%; heat-curing PMMA preferred due to lower toxicity; biocompatibility research ongoing [88,89].
Polysulfide Rubber	Elastomeric impressions for complete dentures and long-span restorations	Excellent tear strength and flow, flexible, long working time	Produces water during setting (condensation reaction), leading to poor dimensional stability; unpleasant odor/taste; must be poured immediately; now rarely used.
Condensation Silicone	General elastomeric impressions (crowns, bridges, dentures)	Good elasticity, fine detail reproduction, pleasant odor/taste	Economical but prone to shrinkage due to alcohol byproduct; hydrophobic; requires immediate pouring for accuracy.
Polyether	High-precision impressions for crowns, bridges, and implant cases	Naturally hydrophilic, excellent accuracy, no volatile byproducts, high stiffness	Excellent in moist fields; short working time; may be difficult to remove from undercuts; absorbs water if overexposed; more expensive than alginate or polysulfide.
Polyvinyl Siloxane (PVS)/Addition Silicone	Gold standard for fixed prosthodontics and implant impressions	Excellent dimensional stability (<0.2% shrinkage), 99% elastic recovery, multiple viscosities available [106]	Odorless, tasteless, stable for storage/re-pouring; hydrophobic unless modified with surfactants; latex gloves can inhibit setting; superior accuracy and comfort.
Vinyl Polyether Silicone (VPES)/Hybrid (PVS + Polyether)	Advanced elastomeric material combining PVS stability and polyether wettability	Improved hydrophilicity, tensile strength, and elastic recovery; maintains dimensional accuracy	Allows accurate impressions in moist environments; intermediate flexibility; requires technique awareness; increasingly used in complex restorative cases.

Sources for numerical values in table: [81,83,87,88,89,93,106].

## Data Availability

No new data were created or analyzed in this study.

## Abbreviations

The following abbreviations are used in this manuscript:

CAD	Computer aided design
CAM	Computer aided manufacturing
PMMA	Poly methyl methacrylate
PEEK	Polyether ether ketone
MEDLINE	Medical Literature Analysis and Retrieval System Online
UV	Ultraviolet
PEKKTON	High performance polyether ketone ketone based dental polymer
PEKK	Polyether ketone ketone
GMA	Glycidyl methacrylate
UDMA	Urethane dimethacrylate
PICN	Polymer infiltrated ceramic network
NACP	Nanoparticles of amorphous calcium phosphate
RBS	Resin based sealant
GI	Glass ionomer
RMGI	Resin modified glass ionomer
PCL	Polycaprolactone
PLA	Polylactic acid
PLC	Polycaprolactone lactide copolymer
EV	Extracellular vesicle
TMV	Tobacco mosaic virus
PGA	Polyglycolic acid
PLLA	Poly L lactic acid
PDLA	Poly D lactic acid
PTFE	Polytetrafluoroethylene
MMA	Methyl methacrylate
PVS	Polyvinyl siloxane
VPES	Vinyl polyether silicone
PVES	Polyvinyl ether silicone
RBC	Resin based composite
MDP	Methacryloyloxydecyl dihydrogen phosphate
